# Increasing Vegetable Intake Using Monosodium Glutamate in a Randomized Controlled Trial: A Culinary Medicine Intervention

**DOI:** 10.1002/fsn3.70441

**Published:** 2025-06-17

**Authors:** Carson Maher, Michelle Alcorn, Allison Childress, John A. Dawson, Shannon Galyean

**Affiliations:** ^1^ Department of Nutritional Sciences Texas Tech University Lubbock Texas USA; ^2^ Department of Hospitality and Retail Management Lubbock Texas USA; ^3^ Department of Economics, Applied Statistics, and International Business New Mexico State University Las Cruces New Mexico USA

**Keywords:** culinary medicine, monosodium glutamate, palatability, sensory evaluation, vegetable intake

## Abstract

This study aimed to explore the effectiveness of monosodium glutamate (MSG) as a flavor enhancer in increasing vegetable intake compared to sodium chloride (NaCl) alone combined with a digital culinary medicine education program. A two‐phase randomized controlled trial (RCT) was conducted from February to November 2023. Phase one involved a five‐week intervention where participants received a designated seasoning and logged their vegetable intake using the MyFitnessPal app. Phase two involved a sensory evaluation, assessing the palatability of green beans and sweet potatoes seasoned with NaCl/MSG mixtures using a Likert scale and triangle tests to determine preference and palatability. Phase one; 60 participants were assigned to one of three groups: 100% NaCl (control), 50/50 NaCl/MSG, and 70/30 NaCl/MSG. Phase two; 88 participants and all seasoning groups received a digital culinary medicine education program with recipes and videos. The 50/50 NaCl/MSG group showed a mean increase in vegetable intake from 1.46 to 1.55 cups/day, while the NaCl group showed a decrease from 1.33 to 0.95 cups/day (*p* = 0.46). Preference tests indicated favorability trends for MSG mixtures, particularly with green beans seasoned with the 50/50 NaCl/MSG mixture (*p* = 0.07). Although the differences in vegetable intake were not statistically significant, the findings suggest that MSG could enhance vegetable palatability and intake, aligning with the principles of culinary medicine. This represents a promising strategy for improving dietary habits. Further research is warranted to confirm these findings.

**Trial Registration:** Clinicaltrials.gov identifier: NCT05591612

## Graphical abstract

MSG could enhance vegetable intake, aligning with the principles of culinary medicine. This intervention represents a viable strategy that improves diet quality, offering a harmonious blend of health and palatability.

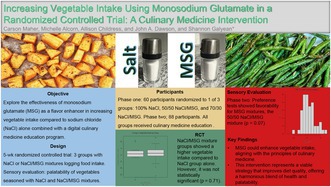

## 1 Introduction

The consumption of vegetables is essential for maintaining health and preventing chronic diseases (Rodriguez‐Casado 2016). Vegetables provide a rich source of vitamins, minerals, fiber, and antioxidants, which are crucial for bodily functions and disease prevention (Latetia V. Moore and Thompson 2015). However, nine out of ten Americans do not meet the recommended daily intake of vegetables (“Only 1 in 10 Adults Get Enough Fruits or Vegetables,” *World Food Regulation Review* 2017; Turnwald et al. 2019). This shortfall is primarily attributed to taste preferences, lack of convenience, and limited knowledge of preparation methods (Kaur 2023).

Including methods to reduce effort will be essential to improve vegetable intake among adults in America. Reducing the effort to achieve and sustain a goal will improve the likelihood of achieving and maintaining that goal (Baumel and Muench 2021). To put it simply, the chances that an individual will take part in desired behaviors (e.g., increasing vegetable consumption) will be boosted by making these behaviors easy and convenient.

The technology industry has shown to reduce the complexity and number of steps needed to reach desired goals (Baumel and Muench 2021). Technology based on culinary medicine provides practical ideas designed for people who ask, “What do I eat for my health?” and can reduce some of the barriers specific to vegetable consumption. In culinary medicine, special attention is given to how the food works in the body as well as the sociocultural and pleasurable aspects of eating and cooking (Irl B et al. 2019). Culinary medicine aims to empower people to care for one's self safely, effectively, and pleasurably with food and beverage (La Puma 2015). Registered dietitians are medical professionals who translate the science of nutrition into practical solutions for everyday living (Bleich et al. 2012; Walker et al. 2012). This can be a helpful strategy to help people eat more vegetables in their daily diets.

Furthermore, excessive sodium intake is correlated with high blood pressure and cardiovascular risks. Therefore, in addition to increasing vegetable consumption, another public health challenge is to reduce sodium intake, which may reduce the palatability of foods. To ease taste barriers for vegetable consumption, monosodium glutamate (MSG) is a flavor enhancer that has been extensively used in the food industry to improve the palatability of various foods (Cobb et al. 2012; T. Wallace et al. 2020). Unlike sodium chloride (NaCl), MSG can enhance the umami flavor without contributing significantly to sodium intake (Maluly et al. 2017). Despite the historical controversy surrounding MSG, studies have indicated its safety and potential benefits in enhancing the palatability of foods, which could lead to increased consumption of nutrient‐dense vegetables (Okiyama and Beauchamp 1998; Yamaguchi and Takahashi 1984; Zanfirescu et al. 2019).

Research has consistently shown that taste preferences play a significant role in determining dietary choices, especially among vegetables (Liem and Russell 2019). While studies have demonstrated that NaCl/MSG mixtures can improve the taste of various foods, including soups, meats, and vegetables (dos Santos et al. 2014; Yamaguchi and Takahashi 1984), there is limited research on its effectiveness in promoting vegetable intake specifically. The purpose of this randomized controlled interventional study is to test the acceptability of vegetables using various NaCl/MSG mixtures as a palatability enhancer combined with culinary medicine education involving cooking demonstrations to encourage the intake of vegetables. To the authors' knowledge, there is no research demonstrating this combination of culinary medicine emphasizing MSG seasonings to enhance the flavor of vegetables. The study aims to identify if vegetables prepared with NaCl/MSG mixtures will increase vegetable consumption compared to those prepared with pure NaCl, and to assess the palatability of these vegetables. This research will aid in expanding knowledge of using NaCl/MSG mixtures as a flavor enhancer to increase vegetable intake and reduce sodium consumption with accessible videos, recipes, and other features to help individuals with meal preparation skills, increased knowledge of recommendations, portions, and health benefits of vegetables.

## 2 Materials and Methods

Participants were recruited through a university‐wide online announcement system reaching all students and faculty. This study was conducted in two phases from February to November 2023. The sample size was calculated based on a similar study in terms of outcome measures and methods, and its cooking education to make a change to diet emphasizing plant‐based foods like vegetables (Carmody et al. 2008). Sixteen participants were needed per group to provide 80% power at a 5% (two‐sided) level of significance. Since there were three groups, a sample size of 48 participants was needed for Phase One. According to Gacula and Rutenbeck, 40–100 participants is an ideal sample size for analytic consumer sensory tests (Gacula and Rutenbeck 2006). Therefore, the aim was to recruit 100 men and women for Phase Two. The study was approved by the Texas Tech University Institutional Review Board (IRB2022‐396), and all participants provided written informed consent before participating in the study.

### 2.1 Study Design

Sixty participants completed baseline study assessments and were randomly assigned to one of three groups: the Control Group (NaCl), consisting of individuals who received pure NaCl to season their vegetables; the 50/50 NaCl/MSG Group, consisting of individuals who received a 50/50 NaCl/MSG mixture to season their vegetables; and the 70/30 NaCl/MSG Group, consisting of individuals who received a 70/30 NaCl/MSG mixture to season their vegetables. Recruitment took place through email correspondence from February to March 2023. Inclusion criteria required all participants to be 18 years or older, interested in increasing their vegetable intake, and in possession of a smartphone capable of downloading apps. Exclusion criteria included allergies to MSG or vegetables and lack of access to a full kitchen.

This study followed a randomized controlled intervention design, with a duration of 5 weeks. Participants underwent a one‐week baseline dietary intake measurement using the MyFitnessPal app, where they logged all food and beverage intake. Participants were then randomized using a block randomization scheme with blocks of size six for the 4‐week intervention. Each group received a specific type of coded seasoning bottle to replace their salt bottle usage on all foods (pure NaCl, 50/50 NaCl/MSG, or 70/30 NaCl/MSG) to ensure a blind study design. The seasoning was weighed pre‐ and post‐intervention to determine salt usage.

During the intervention, participants received cooking demonstrations, recipe handouts, and nutrition education videos twice weekly via email, aimed at increasing vegetable intake. They also continued to log their dietary intake using the MyFitnessPal app throughout the 4 weeks. Weekly tallies of vegetable servings were calculated, and the average vegetable intake during the intervention was compared to the baseline intake to assess changes. Additionally, a Diet History Questionnaire (DHQ‐3) was administered pre‐ and post‐intervention to capture dietary patterns.

### 2.2 Sensory Evaluation

Eighty‐eight adults participated in the sensory evaluation phase, which included preference and triangle tests to assess the palatability of green beans and sweet potatoes seasoned with either NaCl/MSG mixtures or NaCl alone. Recruitment began in April 2023 and concluded in November 2023. Participants underwent a one‐time sensory evaluation where they assessed the sensory characteristics of the vegetables (preference test) or their ability to distinguish between different seasonings (triangle test). Inclusion criteria for this phase required participants to be 18 years or older and willing to consume vegetables seasoned with MSG. Exclusion criteria included allergies to MSG or vegetables.

Frozen green beans (half to one inch in length) were mixed with olive oil and seasoning, then cooked in a skillet until tender‐crisp and maintained on low heat. The beans were refreshed every 45 min to ensure consistent quality. Pre‐cut sweet potato cubes were mixed with olive oil and seasoning, then roasted at 400°F for 30–45 min and held in an electric, water‐based chafer dish for serving.

The preference test used the green beans and sweet potatoes seasoned with blinded 50/50 and 70/30 NaCl/MSG mixtures and pure NaCl. Participants answered questions on overall liking, appearance, texture, flavor, saltiness, and willingness to cook/eat again, using a 1–9 Likert scale, where 1 indicated “dislike extremely” and 9 indicated “like extremely.” The triangle test used the green beans to determine if participants could detect differences between the blinded NaCl/MSG mixtures and pure NaCl. Participants identified the odd sample out of three, with confidence levels rated from “1” (not confident) to “5” (completely confident). No significant changes to the trial methods were made after commencement. Any minor adjustments were administrative and did not impact the study's design, procedures, or outcomes.

### 2.3 Statistical Analysis

Outcome measures included changes in vegetable intake, sensory evaluation scores, and salt usage. No changes were made to the primary or secondary outcomes after the trial commenced. All outcomes were measured as initially planned. The random allocation sequence was generated using a computer‐based random number generator to ensure randomization integrity. The sequence was created prior to the start of participant recruitment and was stratified by baseline vegetable intake levels. Paired t‐tests were used to evaluate changes in vegetable intake and DHQ‐3 questionnaire results. Vegetable palatability changes were assessed using Wilcoxon tests. A generalized linear mixed‐effects model was used for the triangle test analysis. Participants who failed to complete more than 50% of the MyFitnessPal diary intake or met an exclusion criterion post‐enrollment were excluded from the final analysis. Statistical analyses were performed using R version 4.2.1.

A block randomization method was employed with blocks of size six to maintain balance across the intervention groups. This approach ensured an equal distribution of participants in each group throughout the trial. The random allocation sequence and assignment to intervention groups were implemented by an independent researcher not involved in the recruitment or intervention phases. Allocation was concealed from the study coordinators until participants had completed the baseline assessments to prevent selection bias.

Participants and outcome assessors were blinded to the group assignments to minimize bias. Seasoning bottles were coded and provided to participants without any indication of their contents. Outcome data were collected and analyzed by blinded assessors. All seasoning bottles were identical in appearance and labeling, differing only in their contents (pure NaCl, 50/50 NaCl/MSG, or 70/30 NaCl/MSG). This ensured that participants could not distinguish between different intervention groups based on the seasoning bottles.

## 3 Results

### 3.1 Phase One Recruitment and Demographics

**FIGURE 1:**
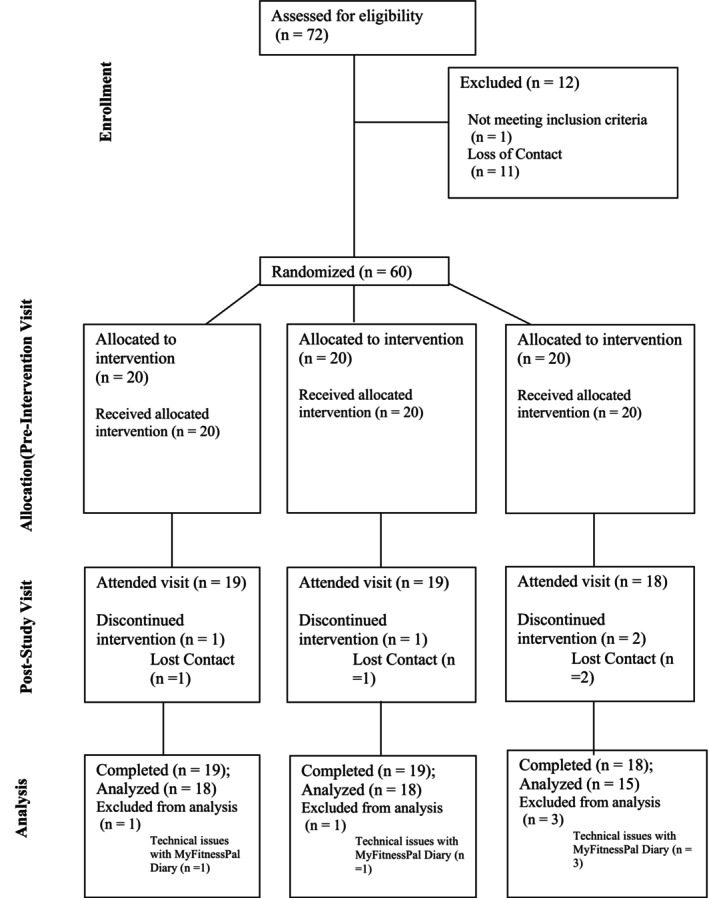
Participants' CONSORT (Consolidated Standards of Reporting Trials) flow diagram for vegetable intake phase.

Initially, 72 participants expressed interest in the study. After the online questionnaire screening phase, 12 participants (16.7%) were excluded for not meeting the inclusion criteria or due to loss of contact. Consequently, 60 participants (Table 1) were deemed eligible and randomized into three groups: 20 participants in the 50/50 NaCl/MSG group, 20 in the 70/30 NaCl/MSG group, and 20 in the pure NaCl group. During the study, 9 participants (15% of the eligible participants) withdrew, dropped out, or failed to complete their vegetable log on MyFitnessPal. The final participant count for data analysis was 18 in the 50/50 NaCl/MSG group, 18 in the 70/30 NaCl/MSG group, and 15 in the pure NaCl group (see Figure 1).

**TABLE 1:** Demographics of phase one participants (vegetable intake phase).

Variable^a^	All groups (*n* = 60)
*Participant characteristics*	
Sex, *n*	
Male	24
Female	36
Age, med [IQR]	22 [20, 24]
Ethnicity, *n*	
African American	2
Asian	31
Caucasian	15
Latino/Hispanic	10
Two/more	1
Other/unknown	1

^a^ Randomized controlled trial (February 2023–April 2023); Texas Tech University Nutrition and Metabolic Health Initiative) evaluating the effect of sodium chloride/monosodium glutamate mixtures on vegetable intake.

#### 3.1.1 Change in Vegetable Intake

**FIGURE 2:**
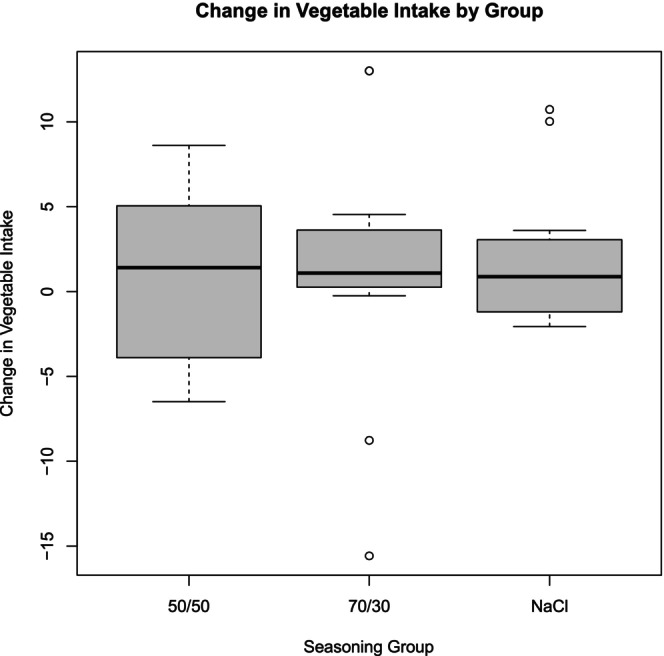
Change in vegetable intake (servings) by group. The figure illustrates the mean change in vegetable intake (servings) for the NaCl, 50/50 NaCl/MSG, and 70/30 NaCl/MSG groups. The 50/50 MSG group showed the highest increase in vegetable intake, followed by the 70/30 MSG group, while the NaCl group had the lowest intake. The boxes represent the interquartile range (IQR), with the dark line inside each box indicating the median change in vegetable intake. Whiskers extend to 1.5 times the IQR, representing the range of the data, excluding outliers. Hollow dots denote outliers, highlighting significant deviations in vegetable intake. The 50/50 seasoning group shows the greatest variability and number of outliers, while the 70/30 group exhibits the least variability.

**FIGURE 3:**
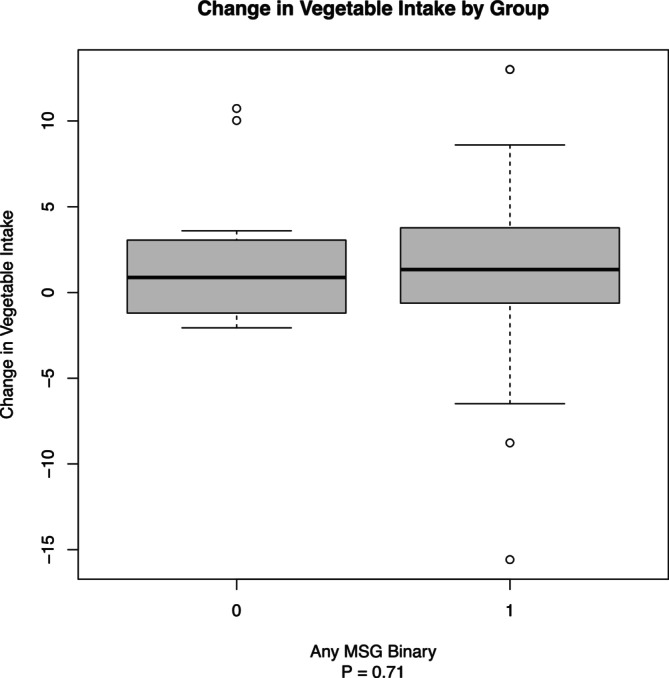
Change in vegetable intake (servings) by group. Box plot showing the change in vegetable intake by the presence of monosodium glutamate (MSG) in seasoning (0 = no MSG, 1 = MSG). The boxes represent the interquartile range (IQR), with the dark line inside each box indicating the median change in vegetable intake. Whiskers extend to 1.5 times the IQR, representing the range of the data, excluding outliers. Hollow dots denote outliers, highlighting significant deviations in vegetable intake (*p* = 0.71).

The analysis of vegetable intake among the different seasoning groups revealed nuanced differences. The 50/50 NaCl/MSG group exhibited the highest mean intake of vegetables, followed closely by the 70/30 NaCl/MSG group. The pure NaCl group had the lowest mean intake (see Figure 2). When combining the NaCl/MSG mixture groups for analysis, a higher mean vegetable intake was observed compared to the NaCl group alone. However, this difference did not reach statistical significance (*p* = 0.71) based on a *t*‐test with multiple imputation (Team 2022; van Buuren and Groothuis‐Oudshoorn 2011). This suggests a trend toward increased vegetable intake with NaCl/MSG mixtures, but the effect was not definitive within the study's parameters. Outliers, represented as hollow dots, were from individuals with high‐calorie diets (see Figure 3). The DHQ‐3 data supported the MyFitnessPal data, showing changes in daily vegetable consumption from baseline to post‐intervention. Despite these changes, they were not statistically significant (*p* = 0.462) (see Table 2).

**TABLE 2:** Diet history questionnaire 3 (DHQ‐3) vegetable intake (cups/day) change, baseline vs. post‐intervention.

Variable^a^	50/50^b^	70/30^c^	NaCl^d^
Baseline	1.46	1.01	1.33
Post‐intervention	1.55	1.01	0.95
Change	+0.11	0.00	−0.38

^a^ Randomized controlled trial (February 2023–April 2023); Texas Tech University Nutrition and Metabolic Health Initiative) evaluating the effect of sodium chloride/monosodium glutamate mixtures on vegetable intake.

^b^ 50% Sodium chloride and 50% monosodium glutamate mixture.

^c^ 70% Sodium chloride and 30% monosodium glutamate mixture.

^d^ 100% Sodium chloride.

#### 3.1.2 Seasoning Use

**FIGURE 4:**
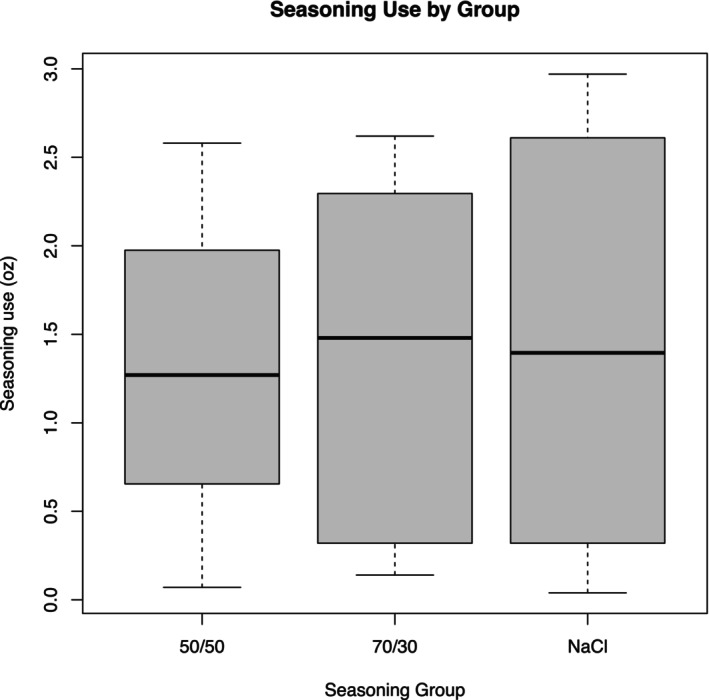
Seasoning use by group. Box plot showing seasoning use (in ounces) by seasoning group (50/50, 70/30, NaCl). The boxes represent the interquartile range (IQR), with the dark line inside each box indicating the median seasoning use. Whiskers extend to 1.5 times the IQR, representing the range of the data, excluding outliers. The plot reveals that the NaCl group has the highest median seasoning use, followed by the 70/30 group and the 50/50 group. This box plot provides insights into the distribution and variability of seasoning use across the different groups.

**FIGURE 5:**
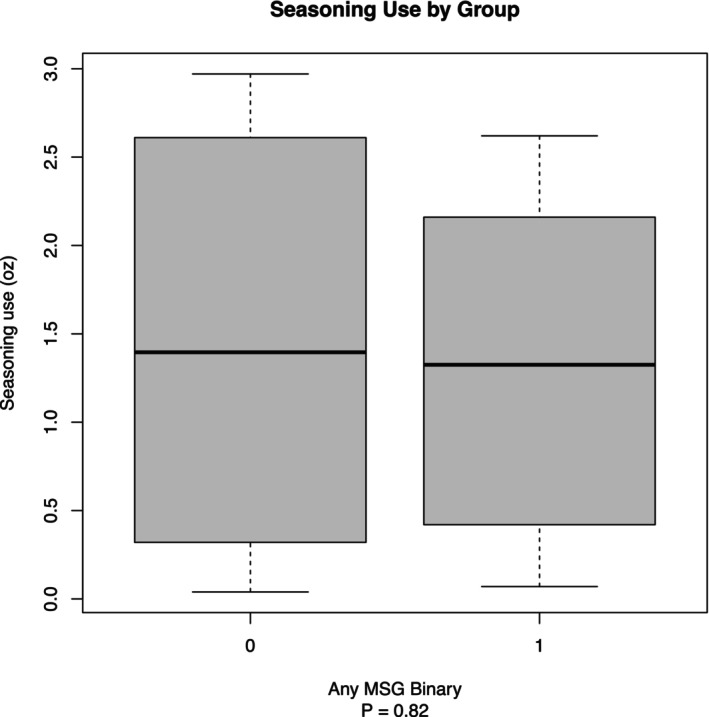
Seasoning use by group. Box plot showing seasoning use (in ounces) by the presence of monosodium glutamate (MSG) in seasoning (0 = no MSG, 1 = MSG). The boxes represent the interquartile range (IQR), with the dark line inside each box indicating the median seasoning use. Whiskers extend to 1.5 times the IQR, representing the range of the data, excluding outliers. The plot demonstrates that both groups (with and without MSG) have similar median seasoning use, with the group without MSG showing slightly higher variability (*p* = 0.82).

Figure 4 shows the analysis of seasoning use demonstrating varying levels of salt consumption among the groups. There was no significant difference in seasoning usage between the MSG mixtures and NaCl alone. The 70/30 NaCl/MSG mixture group used the highest amount of seasoning, followed by the NaCl group, with the 50/50 NaCl/MSG mixture group using the least amount (*p* = 0.82). When consolidating the 50/50 and 70/30 NaCl/MSG mixture groups into a single category, the combined NaCl/MSG mixture group exhibited lower average salt usage compared to the NaCl‐only group. This comparison yielded a *p* value of 0.82, indicating no significant difference in salt consumption behavior following the introduction of MSG into the seasoning mixtures (see Figure 5).

### 3.2 Phase Two Preference and Triangle Tests

**FIGURE 6:**
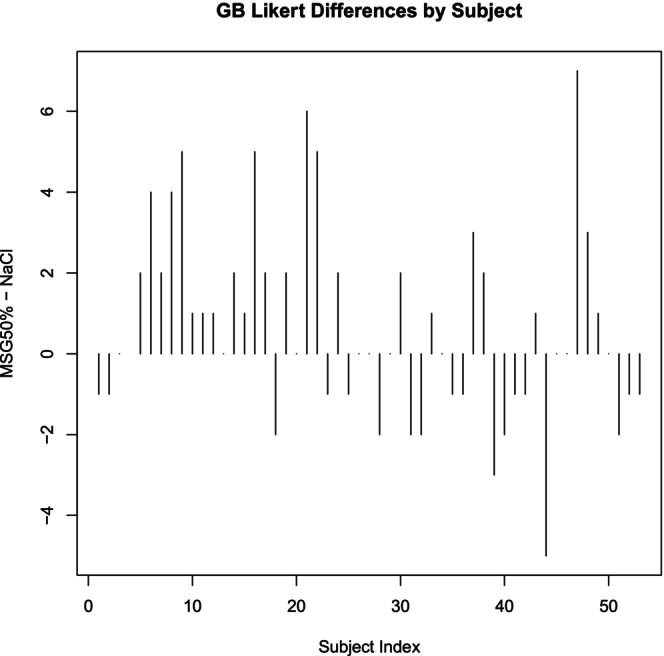
Green bean (GB) bar plot Likert differences by participant. Bar plot showing the differences in GB Likert scores by subject between the MSG 50% and NaCl groups. Each bar represents the difference in GB Likert scores for an individual subject (MSG 50% − NaCl). Positive values indicate a higher score in the MSG 50% group compared to the NaCl group, while negative values indicate a lower score. The plot provides a subject‐wise comparison of the perceived effects of the two seasoning types, highlighting the variability in individual responses.

**FIGURE 7:**
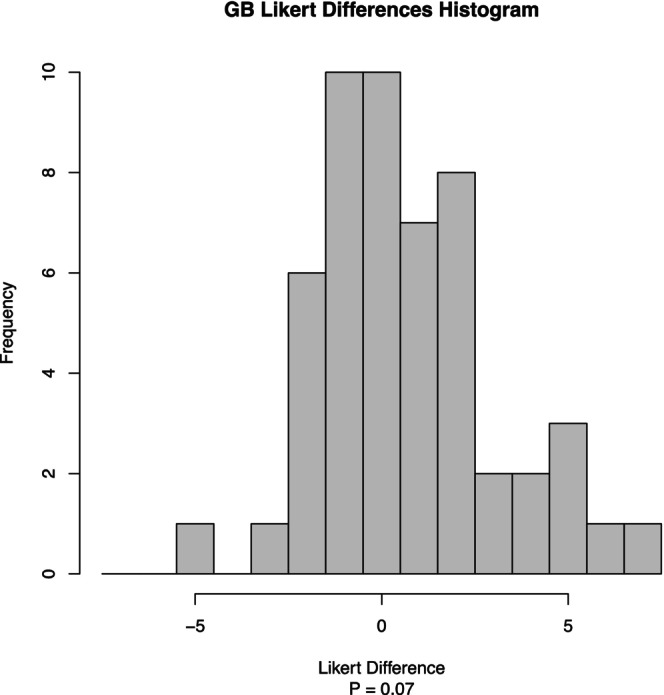
Green bean histogram Likert differences by frequency. Histogram showing the distribution of GB Likert score differences between the MSG 50% and NaCl groups. Each bar represents the frequency of subjects with a particular Likert score difference (MSG 50% − NaCl). Positive values indicate higher scores in the MSG 50% group compared to the NaCl group, while negative values indicate lower scores (*p* = 0.07).

**FIGURE 8:**
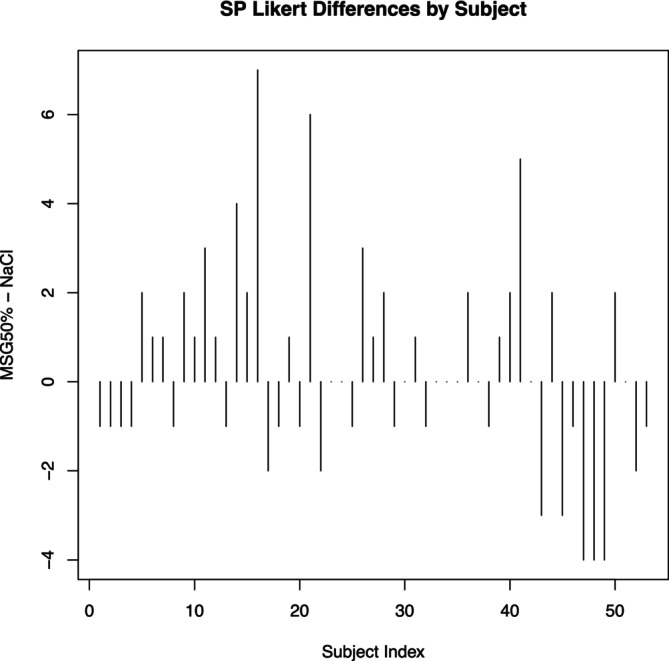
Sweet potato (SP) bar plot Likert differences by participant. Bar plot showing the differences in SP Likert scores by subject between the MSG 50% and NaCl groups. Each bar represents the difference in SP Likert scores for an individual subject (MSG 50% − NaCl). Positive values indicate a higher score in the MSG 50% group compared to the NaCl group, while negative values indicate a lower score. The plot provides a subject‐wise comparison of the perceived effects of the two seasoning types, highlighting the variability in individual responses.

**FIGURE 9:**
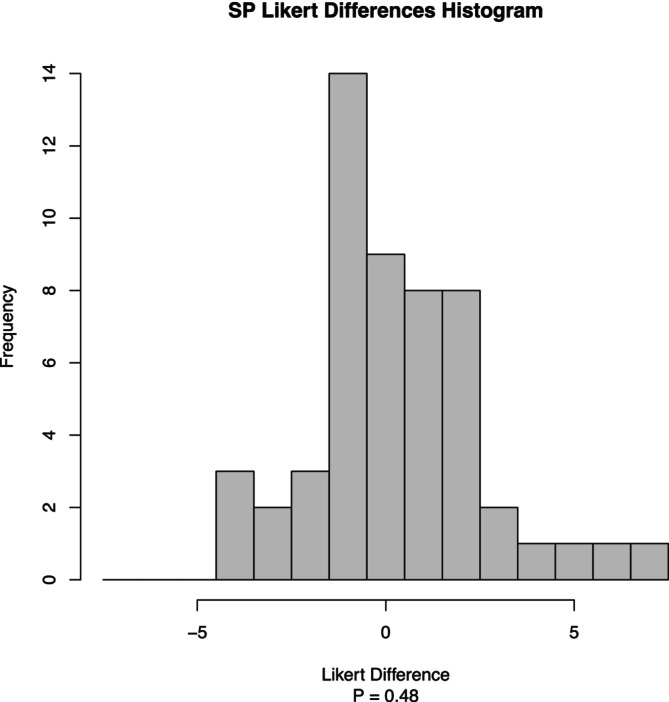
Sweet potato histogram Likert differences by frequency. Histogram showing the distribution of SP Likert score differences between the MSG 50% and NaCl groups. Each bar represents the frequency of subjects with a particular Likert score difference (MSG 50% − NaCl). Positive values indicate higher scores in the MSG 50% group compared to the NaCl group, while negative values indicate lower scores (*p* = 0.48).

Phase two enrolled 96 participants (62 from the Preference Test and 34 from the Triangle Test). See Tables 3 and 4. The Preference Test compared the 50/50 NaCl/MSG mixture against the pure NaCl mixture. Analysis revealed a general preference for the 50/50 NaCl/MSG mixture in green beans and sweet potatoes, though not statistically significant. The Likert difference scores for green beans (Figures 6 and 7) show a positive trend toward the 50/50 NaCl/MSG mixture (*p* = 0.07). Similarly, the results for sweet potatoes (Figures 8 and 9) indicate a preference for the 50/50 NaCl/MSG mixture, but without statistical significance (*p* = 0.48). The Triangle Test assessed participants' ability to correctly identify the odd sample among three samples (see Table 5). Overall, the study's results suggest that the 50/50 or 70/30 NaCl/MSG mixtures can enhance vegetable intake and improve palatability without significantly increasing salt consumption. However, further research is needed to confirm these findings and explore the potential health implications.

**TABLE 3:** Green bean and sweet potato preference test demographics for sensory evaluation phase.

Variable^a^	All groups (*n* = 62)
*Participant characteristics*	
Sex, *n*	
Male	15
Female	37
Age, med [IQR]	21 [19, 31]
Race and Ethnicity, *n*	
African American	2
Asian	1
Caucasian	37
Latino/Hispanic	6
Two/more	4
Other/unknown	3
Degree, *n*	
High school	31
Bachelors	10
Masters	9
Phd/higher	2
Prefer not to say	1
Marital Status, *n*	
Single	40
Divorced	3
Married	10
Annual Income (MED) [IQR]	$66k [$36k, $130k]
Employment, *n*	
Full‐time	20
Seeking opportunities	4
Not employed	11
Part‐time	18

^a^ Randomized controlled trial (May 2023–November 2023); Texas Tech University Nutrition and Metabolic Health Initiative) and the Department of Nutritional Sciences Culinary Lab, evaluating the effect of sodium chloride/monosodium glutamate mixtures on green bean and Sweet Potato palatability.

**TABLE 4:** Green bean triangle test demographics for sensory evaluation phase.

Variable^a^	All groups (*n* = 62)
*Participant characteristics*	
Sex, *n*	
Male	11
Female	23
Age, med [IQR]	30 [24, 40]
Race and Ethnicity, *n*	
African American	3
Asian	11
Caucasian	12
Latino/Hispanic	4
Two/more	2
Other/unknown	1
Degree, *n*	
High school	7
Bachelors	7
Masters	16
Phd/higher	4
Prefer not to say	1
Marital Status, *n*	
Single	20
Divorced	2
Married	11
Annual income (MED) [IQR]	$38.5k [$23k, $100k]
Employment, *n*	
Full‐time	12
Seeking opportunities	3
Not employed	4
Part‐time	15

^a^ Randomized controlled trial (May 2023–November 2023); Texas Tech University Nutrition and Metabolic Health Initiative) and the Department of Nutritional Sciences Culinary Lab, evaluating the effect of sodium chloride/monosodium glutamate mixtures on Green Bean palatability.

**TABLE 5:** Green bean triangle test confidence levels with correct and incorrect guesses^a^.

Variable^b^	1 Confidence (*n*)	2 Confidence (*n*)	3 Confidence (*n*)	4 Confidence (*n*)	5 Confidence (*n*)
Incorrect	4	7	18	27	7
Correct	3	3	11	7	16

^a^ Triangle Test Confidences. The table shows the distribution of confidence levels among participants for correct and incorrect guesses in the Triangle Test. The results reveal that confidence levels did not correlate with guessing accuracy, as 40 out of 104 trial rounds were correct, aligning closely with random chance (*p* = 0.30). Gender differences in guessing performance were not significant.

^b^ Randomized controlled trial (May 2023–November 2023; Texas Tech University Nutrition and Metabolic Health Initiative) and the Department of Nutritional Sciences Culinary Lab, evaluating the effect of sodium chloride/monosodium glutamate mixtures on vegetable palatability.

## 4 Discussion

This randomized controlled interventional study aimed to compare vegetables prepared with NaCl/MSG mixtures to those prepared with pure NaCl, to identify if the former can significantly increase vegetable intake and their palatability compared to vegetables seasoned with pure NaCl. This study is very novel in its approach for many reasons: First, it targets the use of NaCl/MSG mixtures in a randomized controlled study to boost vegetable consumption, leveraging MSG's umami flavor to address under‐consumption of vegetables—unlike its common use in soups or meats (Yamaguchi and Takahashi 1984). Secondly, the study blends culinary medicine nutrition and cooking skills into an RCT framework making it a practical approach that enhances dietary interventions, cooking skills, and practical education beyond traditional nutritional education methods. On the other hand, previous studies used pre‐seasoned foods that the participants did not prepare in their own home and therefore lacked generalizability (Turnwald et al. 2019). Third, it had a dual‐purpose strategy that focused on vegetable palatability and sodium reduction, unlike broader sodium‐reduction studies where other dishes were targeted (Jinap et al. 2016). Lastly, the study incorporated a combined quantitative and qualitative design assessing MSG's impact on vegetable intake (Phase 1) and palatability (Phase 2). This comprehensive approach provides richer insights than single‐phase studies (Halim et al. 2020).

Phase one results demonstrated that using NaCl/MSG mixtures slightly increased vegetable intake compared to NaCl alone, with the highest intake observed in the 50/50 NaCl/MSG group. The DHQ‐3 data corroborated the MyFitnessPal findings, indicating an increase in vegetable consumption in the 50/50 group, though these differences were not statistically significant, suggesting a limited effect of MSG on enhancing vegetable consumption (Jinap and Hajeb 2010). The findings indicate that while NaCl/MSG mixtures may influence vegetable intake and seasoning behavior, their impact is not distinctly different from using NaCl alone. Salt usage varied among groups, with the 70/30 NaCl/MSG mixture showing the highest usage, but again, differences between MSG mixtures and NaCl alone were not significant. This suggests that the benefits of MSG in promoting vegetable consumption or reducing salt use are minimal, requiring further research to assess their effectiveness in dietary improvements.

Phase two results indicated a preference for the NaCl/MSG mixtures, with slight preferences for the 50/50 NaCl/MSG mixture over pure NaCl in seasoning green beans and sweet potatoes, although not statistically significant. The findings suggest the MSG mixtures ability to enhance vegetable palatability and intake, aligning with existing literature on MSG's flavor enhancement capabilities. Previous studies, such as Okiyama et al. found that adding MSG to chicken broth increased its palatability at low to moderate salt levels. The study demonstrated that both components of MSG—sodium and glutamate—individually contributed to taste enhancement, with subjects preferring soups with added sodium at lower salt concentrations and those with added glutamate at moderate salt levels, which demonstrates the distinct roles of sodium and glutamate in improving flavor (Okiyama and Beauchamp 1998).

Comparing our findings with existing literature, particularly the study by Okiyama et al. underscores the importance of the right seasoning balance for vegetable intake. Our study demonstrated a pronounced preference for vegetables seasoned with a 50/50 NaCl/MSG ratio. This pattern aligns with the preference for MSG‐enhanced broth at lower salt concentrations in Okiyama et al.'s work (Okiyama and Beauchamp 1998). This parallel suggests that an optimal mix of NaCl and MSG significantly boosts the palatability of vegetables, enhancing overall consumption. The 70/30 NaCl/MSG ratio, although less effective than the 50/50 mix, still surpassed pure NaCl in appeal, supporting the idea that MSG can make vegetables more appetizing even at lower concentrations. The diminished vegetable intake observed with pure NaCl seasoning reinforces MSG's potential as a pivotal palatability enhancer. These findings collectively highlight MSG's role in dietary strategies aimed at elevating vegetable consumption while concurrently managing sodium intake—affirming its value in promoting healthier eating habits.

Limited research has been conducted on the impact of combining NaCl and MSG into one seasoning on the consumption of vegetables. Halim et al. explored healthy food options, like vegetables, but they did not specifically focus on vegetable consumption. Their findings highlighted a general preference for MSG‐enhanced recipes, with 68% of participants favoring them equally or more than those with reduced or regular sodium levels, finding that the MSG‐enhanced savory dip was significantly more liked than the standard and reduced salt dip (*p* < 0.05) (Halim et al. 2020). Comparing our findings to this study, our results contribute nuanced insights into the specific context of vegetable intake. While we hypothesized that MSG seasoning would lead to an increase in vegetable consumption compared to pure NaCl seasoning, this was not supported by our data as there was no significant difference in intake between the MSG and NaCl groups (*p* = 0.71). However, within the MSG groupings, the 50/50 NaCl/MSG ratio emerged as the most favored, pointing to a preference for a balanced mix of MSG and salt in making vegetables more appealing. The 70/30 ratio, though not as effective as the 50/50, still surpassed the appeal of vegetables seasoned solely with NaCl. This preference pattern suggests that even subtle variations in MSG and NaCl proportions can influence the palatability of vegetables. Halim et al.'s findings, which did not focus on vegetable consumption per se but highlighted the potential of MSG to enhance dietary choices, are relevant. Our study extends this line of inquiry by demonstrating that while MSG may not significantly increase overall vegetable intake, specific NaCl/MSG ratios can enhance the appeal of vegetables, making MSG a valuable tool in dietary interventions promoting vegetable consumption. This suggests that the palatability enhancement afforded by MSG could be a valuable tool in dietary interventions aimed at promoting vegetable consumption.

In another study by Maluly et al. which spanned various food categories such as dairy, meat, and soups, it was shown that the replacement of sodium with umami compounds does not compromise saltiness perception and contributes to public health and safety (Maluly et al. 2017). These studies highlight the lack of research directly linking MSG use to vegetable intake, as studies examining the relationship between vegetable intake and MSG's acceptability are scarce. While our study found no statistically significant increase in vegetable intake with MSG seasoning compared to traditional NaCl seasoning, the preference trends observed for the 50/50 NaCl/MSG ratio underscore the potential of MSG to enhance the appeal of vegetables. This insight aligns with the findings from Maluly et al. which demonstrated that replacing sodium with umami enhancers could maintain or even enhance flavor without compromising health. This suggests a promising avenue for public health strategies aimed at reducing sodium intake, with our research adding evidence linking MSG use and increased vegetable consumption to influence dietary choices. The results of this study help to understand the relationship between vegetable intake and seasoning usage. Further studies are encouraged to delve deeper into this relationship, potentially uncovering new strategies for improving dietary patterns and promoting healthier eating habits.

Beforehand, it was hypothesized that seasoning usage would be higher in the MSG groups compared to the NaCl group. This hypothesis was rejected as the findings indicated that the 70/30 NaCl/MSG mixture group used the highest amount of seasoning, followed by the NaCl group, with the 50/50 NaCl/MSG mixture group using the least amount (*p* = 0.82). The lack of clarity with the MSG groups finishing 1st and 3rd, along with no significant difference in seasoning usage between the MSG mixtures and NaCl alone, suggests that the presence of MSG does not definitively lead to an increase in seasoning usage compared to the use of pure NaCl. As stated above, there are no other published studies that examine NaCl/MSG usage in vegetables found in the literature that are closer to our objectives than Maluly et al. and Halim et al. There are many studies on MSG, but these studies focus on the general formula of MSG to food weight ratio to avoid using too much MSG (Jinap and Hajeb 2010) or studies showing the umami flavor of MSG adding a more complex and satisfying taste experience to food (T. C. Wallace et al. 2019).

The Likert score analysis suggests that participants generally preferred vegetables seasoned with NaCl/MSG mixtures to those seasoned with pure NaCl. This preference indicates a potential for MSG mixtures to enhance the palatability of vegetables, which could be a valuable strategy for increasing vegetable intake. Despite the lack of statistical significance, the observed trend towards favorability for MSG mixtures points to the malleability of taste preferences and the possibility of using MSG as a tool to improve dietary patterns (Liem and Russell 2019). Introducing a new seasoning, like MSG, to the participant can lead someone to either dislike or embrace the change depending on their personal taste preferences. As seen in Leite et al. many people accept new seasonings and even appreciate a perception of a new seasoning in their food (MarinaLeite et al. 2017). The findings underscore the importance of further research to explore how NaCl and MSG mixtures can be optimized to encourage healthier eating habits.

Overall, the findings of the study appear to prefer the 50/50 NaCl/MSG ratio. In Jinap et al. they determined that the ideal combination of NaCl and MSG for maximum acceptance was found to be 0.3% NaCl and 0.7% MSG. This mix allowed for a 32.5% reduction in sodium levels without compromising the overall acceptability. The research indicated that the ratio of NaCl to MSG could reach as high as 70% MSG to 30% NaCl without negatively impacting the taste and acceptance of the food (Jinap et al. 2016). However, in our investigation, after consultation with an executive chef, we opted for a 50% NaCl to 50% MSG ratio. The chef advised that, based on culinary expertise, a higher MSG content might lead to decreased acceptance among consumers. This counsel is substantiated by Leite et al. who found that substituting up to 50% of NaCl with MSG in fish products does not detract from their sensory appeal, yet substituting above 50% of NaCl with MSG altered the sensory perception and hedonic preferences of fish products (MarinaLeite et al. 2017). We chose to examine the 70/30 NaCl/MSG ratio to determine if it would be more attractive than the 50/50 ratio. Our findings indicated that its appeal was comparable to the 50/50 mix. From these results, we concluded that the 50/50 ratio might be more desirable as it offers a further reduction in sodium intake compared to the 70/30 NaCl to MSG ratio.

In conclusion, vegetable intake managed to increase across all seasoning groups. All seasoning groups received culinary medicine recipes, recipe videos, and nutrition education as part of their general study protocol. As seen in Stauber et al. participation in culinary medicine courses was linked to higher adherence to the Mediterranean Diet and enhanced understanding of healthy eating practices (Stauber et al. 2022). Among 1381 participants, cooking classes significantly enhanced Mediterranean Diet adherence by 0.62 points on a 9‐point scale (95% CI 0.23–1.00, *p* = 0.002) and increased the probability of meeting dietary recommendations for fruits (OR 2.77, 95% CI 1.46–5.23, *p* = 0.002), vegetables (OR 4.61, 95% CI 1.85–11.53, *p* = 0.001), legumes (OR 2.48, 95% CI 1.45–4.26, *p* = 0.001), and olive oil use (OR 2.87, 95% CI 1.44–5.74, *p* = 0.003), while reducing perceptions of cooking as time‐consuming (OR 0.31, 95% CI 0.16–0.59, *p* < 0.001). These findings underscore the effectiveness of hands‐on culinary education in promoting healthier eating habits and addressing obesity (Stauber et al. 2022). These interventions offer not only an effective but also an economical solution for increasing intake and therefore reducing a variety of chronic diseases via consuming vegetables.

## 5 Implications

The results of this study have significant implications for various audiences. For clinicians, the findings suggest that using MSG as a seasoning can be a practical approach to promote higher vegetable intake among patients, particularly those who are sensitive to the sodium content of their diet. By incorporating MSG, clinicians can recommend a flavor‐enhancing option that reduces sodium intake while potentially increasing vegetable consumption. For the general public, especially those striving to adopt healthier eating habits, the study provides evidence that MSG can make vegetables more palatable without the adverse effects associated with high sodium intake. This insight can help individuals make informed choices about their seasoning preferences, leading to better adherence to dietary recommendations. For researchers, these findings open up new avenues for exploring the impact of various seasoning mixtures on dietary habits and health outcomes. Further research can build on this study to investigate long‐term effects, different demographic responses, and the broader applicability of MSG in different culinary contexts. Various vegetables can be investigated along with various amounts and ratios of the NaCl/MSG ratios on specific vegetables. For community programs focused on nutrition and public health, the results suggest that incorporating MSG into educational materials and cooking classes could be a viable strategy to improve vegetable intake among participants. Community health initiatives can leverage these findings to design interventions that encourage the use of MSG as a healthier alternative to NaCl, thus promoting better dietary habits at the community level.

This study's exploration into MSG as a substitute for NaCl highlights the potential health benefits of sodium reduction. Excessive sodium intake is associated with increased blood pressure and a higher risk of cardiovascular diseases. By demonstrating that MSG can enhance the flavor of vegetables with significantly less sodium, this study supports public health recommendations for sodium reduction. Clinicians can use these findings to make specific recommendations for patients. For instance, they might suggest using a 70/30 MSG/NaCl mixture to patients looking to reduce their sodium intake without sacrificing taste. Additionally, clinicians may find a digital culinary medicine education program to encourage patients on how to incorporate more vegetables into their diet. This practical advice can help patients integrate healthier seasoning options into their daily diets, potentially leading to better long‐term health outcomes.

## 6 Limitations

The study faced certain limitations that may affect the generalization of its findings. Primarily, the sample size and the specific demographic of students used in the study might not reflect the broader population's dietary habits and responses to NaCl/MSG seasoning. Such constraints suggest that future research should include a more diverse demographic to enhance the applicability of the results across different population segments. We did not have an equal amount of duration for the baseline and the intervention in phase one of the study. The baseline measurement period was only 1 week, whereas the intervention lasted for 4 weeks. This discrepancy made it challenging to accurately compare baseline vegetable intake with the 4‐week intervention period. The short baseline measurement period (1 week) may not have provided a comprehensive understanding of participants' usual vegetable intake. Extending the baseline period could yield more accurate baseline data for future studies to more precisely determine changes in vegetable intake. Additionally, the reliance on self‐reported dietary intake through the MyFitnessPal app may introduce reporting biases. Future research could benefit from more objective measures of dietary intake. Another limitation is that we only used green beans and sweet potatoes for the preference test. These vegetables showed differences in palatability with the three different seasonings, suggesting that the results may not be generalizable to all vegetables. Including a broader range of vegetables in future studies would enhance the generalizability of the findings to more diverse vegetables. There was a limitation in not accounting for potential variations in taste perception and vegetable preference across different ethnic and gender groups. Future research should consider these demographic factors, as cultural and physiological differences may influence the effectiveness of MSG in reducing sodium intake and improving vegetable consumption. Finally, the Jinap et al. study shows that a 30/70 NaCl/MSG can be effective, which we did not use. Future research should explore a variety of NaCl/MSG mixtures, including the 30/70 NaCl/MSG ratio, to determine the optimal balance for reducing sodium intake while maintaining palatability.

## 7 Conclusions

This study explored the impact of NaCl/MSG mixtures on vegetable intake, aiming to uncover palatable alternatives to pure NaCl seasoning that could enhance vegetable consumption. Through careful experimentation and analysis, we observed a discernible preference for vegetables seasoned with a 50/50 NaCl/MSG mixture, suggesting an improved palatability compared to those seasoned solely with pure NaCl. Although the differences in vegetable intake between the NaCl/MSG mixtures and pure NaCl were not statistically significant, the trend indicates a promising direction for dietary modifications to increase vegetable consumption. The findings emphasize the potential of culinary strategies, specifically the strategic use of seasoning mixtures, in promoting healthier eating habits. By demonstrating that NaCl/MSG mixtures can make vegetables more appealing while reducing sodium intake, this research contributes to the broader dialogue on dietary interventions for better health outcomes. It aligns with the principles of culinary medicine, emphasizing the importance of taste and enjoyment in achieving and maintaining dietary changes that can lead to improved health and well‐being. In conclusion, the use of NaCl/MSG mixtures to enhance vegetable intake represents a viable strategy to improve diet quality, offering a harmonious blend of health and palatability.

## Author Contributions

**Carson Maher:** conceptualization (equal), data curation (equal), investigation (equal), methodology (equal), project administration (equal), resources (equal), validation (equal), visualization (equal), writing – original draft (lead), writing – review and editing (equal). **Michelle Alcorn:** conceptualization (equal), methodology (equal), software (equal), visualization (equal), writing – review and editing (equal). **Allison Childress:** conceptualization (equal), methodology (equal), software (equal), visualization (equal), writing – review and editing (equal). **John A. Dawson:** formal analysis (equal), methodology (equal), validation (equal), writing – review and editing (equal). **Shannon Galyean:** conceptualization (equal), data curation (equal), funding acquisition (equal), investigation (equal), methodology (equal), project administration (equal), resources (equal), software (equal), supervision (equal), validation (equal), visualization (equal), writing – original draft (equal), writing – review and editing (equal).

## Ethics Statement

The study was conducted in accordance with the Declaration of Helsinki and approved by the Institutional Review Board of Texas Tech University (IRB2022‐396, protocol code IRB2022‐396, approval date 1‐18‐2023).

## Consent

Informed consent was obtained from all subjects involved in the study.

## Conflicts of Interest

The authors declare a conflicts of interest due to Ajinomoto's involvement in the funding and design of this study. Ajinomoto is a company that manufactures and sells MSG products. Their contribution included financial support and assistance in the study design, which could be perceived as influencing the outcomes of the research. Drs. S.G., A.C., and M.A. are owners of 3 CulinaryMed Docs LLC. The electronic platform was used as part of the nutrition education to help participants know how to prepare vegetables. It was provided for all participants. There was no control that did not receive this type of education.

## Data Availability

Data supporting reported results can be found by contacting the corresponding author. Data will be made available upon request.
